# Insects as a Nitrogen Source for Plants 

**DOI:** 10.3390/insects4030413

**Published:** 2013-07-31

**Authors:** Scott W. Behie, Michael J. Bidochka

**Affiliations:** Department of Biological Sciences, Brock University, St. Catharines, Ontario, L2S 3A1, Canada; E-Mail: sb07fh@brocku.ca

**Keywords:** insects, nitrogen, nutrient transfer, fungus, endophyte, insect pathogen

## Abstract

Many plants have evolved adaptations in order to survive in low nitrogen environments. One of the best-known adaptations is that of plant symbiosis with nitrogen-fixing bacteria; this is the major route by which nitrogen is incorporated into plant biomass. A portion of this plant-associated nitrogen is then lost to insects through herbivory, and insects represent a nitrogen reservoir that is generally overlooked in nitrogen cycles. In this review we show three specialized plant adaptations that allow for the recovery of insect nitrogen; that is, plants gaining nitrogen from insects. First, we show specialized adaptations by carnivorous plants in low nitrogen habitats. Insect carnivorous plants such as pitcher plants and sundews (*Nepenthaceae*/*Sarraceniaceae* and *Drosera* respectively) are able to obtain substantial amounts of nitrogen from the insects that they capture. Secondly, numerous plants form associations with mycorrhizal fungi that can provide soluble nitrogen from the soil, some of which may be insect-derived nitrogen, obtained from decaying insects or insect frass. Finally, a specialized group of endophytic, insect-pathogenic fungi (EIPF) provide host plants with insect-derived nitrogen. These soil-inhabiting fungi form a remarkable symbiosis with certain plant species. They can infect a wide range of insect hosts and also form endophytic associations in which they transfer insect-derived nitrogen to the plant. Root colonizing fungi are found in disparate fungal phylogenetic lineages, indicating possible convergent evolutionary strategies between taxa, evolution potentially driven by access to carbon-containing root exudates.

## 1. Introduction

Nitrogen exists in the soil in numerous forms, and these forms can be rapidly converted from inorganic to organic and *vice versa* [1]. However, nitrogen is often a limiting resource for primary producers and this deficit is due, in large part, to the inability of plants to directly utilize atmospheric nitrogen, and their subsequent reliance on microbial nitrogen fixation. As a result, many plant species (e.g., legumes) form symbiotic associations with microorganisms that can fix atmospheric nitrogen that is then made accessible to the plant [2]. Free-living nitrogen-fixing bacteria (e.g., *Azotobacter*) also contribute to the nitrogen flux in the soil, as they are able to release useable nitrogen (nitrate or ammonia) into the soil. Despite the abundance of microbial nitrogen fixation, nitrogen is still a limiting resource, particularly for many plants without nitrogen-fixing symbionts [3]. However, numerous plant adaptations, and novel plant microbe symbioses, have evolved specifically for the acquisition of nitrogen [4,5].

Soil bacteria and fungal symbionts, such as *Rhizobia* and *Glomus*, respectively, are able to provide plants with substantial amounts of inorganic nitrogen per year in natural settings, as well as in commercial agricultural fields [2,6,7]. These plant symbionts are able to convert N_2_ into inorganic nitrogen (e.g., *Rhizobia*) or convert organic nitrogen in the soil to a form that is transferred to plants (e.g., *Glomus*). Organic nitrogen is present in soil ecosystems bound within leaf litter and other plant detritus, as well as in insect cadavers, insect frass, and living insects [8]. This organic nitrogen is then made available to plants through microbial decomposition. Nitrogen can also be made available for plants through atmospheric fixation of nitrogen when lightning strikes convert N_2_ into NO and NO_2_ that is subsequently deposited in soils through precipitation [9]; however, this type of nitrogen fixation is inconsistent over time and space [9].

Despite the many methods by which nitrogen is converted to an inorganic form that can be used by plants, leaching of nitrogen from the soil due to erosion or poor farming practices, biological processes such as denitrification and microbial competition, all act to remove and limit soil nitrogen levels [10,11,12,13].

Many insects are primary herbivores that strip nitrogen from plants; the nitrogen amassed in insects can comprise a substantial amount of the total nitrogen flux in the ecosystem [14]. The current paradigm of the nitrogen cycle represents the flow of nitrogen within an entire ecosystem, yet there are few models that consider the reservoir of nitrogen in insects. On an ecosystem level, insects can be an essential nitrogen source for plants and should not be overlooked. When viewed on a per plant basis, insects potentially become increasingly important as a source of nitrogen. In this review we discuss how the nitrogen reservoir present within insects is an integral part of the soil nitrogen cycle, and the three major adaptations by which insects can be exploited by plants for their nitrogen.

## 2. Nitrogen in the Soil—Why Is It a Deficit?

The majority of soil ecosystems are nitrogen-limited [15], and nitrogen, along with phosphorus, are the nutrients most likely to limit production in terrestrial environments [16,17]. Primary producers require more nitrogen and phosphorus than other nutrients, and nitrogen is energetically expensive to acquire and utilize [18]. Nitrogen has proven to be critical in the overall health of plants, and the introduction of nitrogen into forested areas, grasslands, or tundra has been shown to increase overall plant productivity [19,20,21,22]. Plant photosynthate production is strongly limited by N availability and plants require substantial amounts of N (2%–5% N content by dry weight, compared with 0.3%–0.5% phosphorus content), and well over 50% of leaf N is devoted to photosynthesis [23]. Therefore, the acquisition of soil nitrogen by plants is directly related to their ability to fix carbon via photosynthesis. 

One of the main reasons for the nitrogen deficit in soils is its mobility. When compared to other important soil nutrients, such as phosphorous, nitrogen is more readily lost through leaching to aquatic systems, and is more sensitive to microbial processes, such as denitrification, that remove nitrogen from the soil [16]. Leaching of nitrogen from natural ecosystems can increase as a result of environmental disturbances, such as insect defoliation [24]. For example, increased deforestation by canker worms has been linked to an increase in nitrate levels in forest streams [24]. Over 70% of the total nitrogen inputs into terrestrial ecosystems can be lost due to natural disturbances and microbial processes [16].

Another major reason for the nitrogen deficit is that some nitrogen is immobilized within decaying plant material. Plant litter is high in nitrogen-containing polymers such as lignin, which is recalcitrant to decomposition, and as such, plant detritus contains large quantities of immobilized nitrogen [25]. In this way, organic nitrogen can collect in large quantities in soil ecosystems, but remains unavailable for plant use [26]. Organically bound nitrogen within decaying plant and animal material, and soil organic matter can account for as much as 90% of the nitrogen in surface soils [27].

While it is clear that there are a number of reasons to explain why nitrogen is limiting in soil ecosystems, there remains an inconsistency concerning this lack of nitrogen. If large nitrogen deficits persist in soil ecosystems, how are primary producers able to acquire the nitrogen required for sustained growth and development? Potentially, there are other nitrogen sources that are unaccounted for by the current models of nitrogen cycling; that is, nitrogen sources such as insects.

## 3. Insects as a Nitrogen Reservoir

In this review, we specifically focus on insects as a source of nitrogen for plants. Insects represent a potential nitrogen loss to plants. Insect herbivory causes the loss of plant material and, therefore, a loss of critical nitrogen. Below ground insect herbivory, specifically, has been found to represent a possible significant loss of plant nitrogen, nitrogen that cannot be replaced through atmospheric or bacterial fixation alone [28]. In this regard, insects can be viewed as nitrogen thieves, whereby herbivorous insects incorporate plant nitrogen. Plants are composed mostly of carbohydrates, and the nitrogen content in actively growing plants typically ranges from 3% to 7% by weight [29]; as such, the loss of any plant material can be devastating to overall plant health.

Despite its potentially damaging effect, below ground insect herbivory has been largely ignored in models of the nitrogen cycle. A reason for this may be that nitrogen cycling is viewed primarily on a whole ecosystem level, which ignores the potential nitrogen deficits that exists at the plant level. A large number of studies have focused on plant predation by above ground insect herbivores; however, root-feeding insects can also cause potentially damaging effects to plants [30]. Below ground herbivory represents a loss of nitrogen from the plants roots, not only in the plant material that has been fed upon, but also through increased levels of nitrogen in the root exudate [10], nitrogen that can then be taken up by competing plants in the soil. On an ecosystem level this does not represent a loss of nitrogen; however, there is a specific loss for the individual plant [6]. The loss of plant material due to insect herbivory of plant roots represents a potentially lethal loss of nitrogen to the plant, and total plant biomass can be reduced by as much as 70% [31].

Insects contain approximately 10% nitrogen by weight, primarily in protein and chitin [32]; in a square hectare of temperate ecosystem soil there is approximately 15 g of insect nitrogen below ground and approximately 100–200 g of insect-bound nitrogen above ground [33]. This amount is not insignificant, particularly when compared to microbially fixed nitrogen in the soil whereby microbes can fix approximately 100 g of nitrogen per hectare per year [34].

This below ground loss of nitrogen can however partially be counterbalanced through the incorporation of insect-derived nitrogen back into plants. Plants are able to accomplish this through: (1) direct ingestion of insects; (2) indirect transfer of insect-derived nitrogen (*i.e.*, decomposing insect cadavers or insect frass) through bacterial or fungal ammonification and nitrification, or transfer of this nitrogen through symbiotic mycorrhizal fungi; or (3) a direct transfer of insect-derived nitrogen through a partnership with endophytic insect pathogenic fungi.

## 4. Carnivorous Plants

There are over 600 known species of carnivorous plants, the majority of which thrive in bogs, marshes and rocky outcrops, or other nitrogen-poor areas where sunlight and water are abundant [35]. These plants have evolved the ability to trap and digest insects, which are an excellent source of nitrogen as they contain around 10% nitrogen by mass [32]. Carnivorous plants are able to obtain between 10% and 80% of their total nitrogen from insects, depending on environment and type of trap employed [5].

Found both terrestrially and in fresh water, pitcher plants (*Nepenthacea*, *Sarraceniaceae*), flypaper traps (*Droseraceae*, *Lentibulariaceae*), snap traps (*Droseraceae*), combination traps (*Aldrovanda*) [5], bladder traps (*Utricularia*), and lobster pot traps (*Genlisea*) [36] have evolved to exploit insect nitrogen. These plants use sophisticated traps and lures in order to capture insect prey, such as emitting specific odors or employing displays visible only in the UV spectrum [37,38]. Insects are then enzymatically digested and insect-derived nutrients are metabolized [39]. A number of carnivorous plants, notably *Sarracenia* and *Drosera*, are able to alter their consumption of insects in response to soil nitrogen fluctuations [40]. Insects remain, however, a critical source of nitrogen for all carnivorous plants.

The most famous of these prey capture mechanisms are the snap traps found in Venus flytraps (*Dionaea muscipula*) and waterwheel plants (*Aldrovanda vesiculosa*). These traps consist of two lobed leaves that are able to close around insect prey when the plant senses movement of one of its trigger hairs [41]. One of the most remarkable aspects of the snap trap is that it employs simple memory to ensure it does not close on precipitation or debris [42]. 

Pitcher plants (*Nepenthaceae*, *Sarraceniaceaer*) are able to attract foraging, flying or crawling insects to their liquid-filled pits; once inside the pit, the insect is enzymatically digested [43]. The lip of the pit fall trap is coated with a slippery mucous-like substance secreted by the plant; when an insect lands there, it is unable to escape [44]. There has been some disagreement over whether nectar bribes or visual cues play the key role in attracting insect prey [45]; however, the end result is that plants are able to acquire nitrogen from trapped insects. Carnivorous plants are able to thrive in areas of extremely low nitrogen, a fact that speaks to the enormous potential of insects as a source of nitrogen.

## 5. Insect Cadavers and Insect Frass: Potential Involvement of Mycorrhizal Fungi

### 5.1. Insect Cadavers/Insect Frass

Insect cadavers present in the soil represent a potentially large reservoir of organic nitrogen [46]. Decaying insects are more easily broken down in soil than other organic material, such as leaf litter, and the nitrogen in insect cadavers is therefore more readily available to plants [47]. Insect frass also contains a large amount of organic nitrogen, in the form of uric acid, that can be converted to inorganic forms, such as ammonia and nitrates [48], and there have been a number of studies that have focused on frass deposition by foraging insects such as termites and gypsy moths [49,50]. It has been shown that, in certain forest ecosystems, insect cadavers and frass deposition can represent up to 70% of the nitrogen returning to the soil when insect populations are large [51]. Insect frass may also provide a source of nutrients for microbial decomposers in the soil, which accelerates the decomposition of natural leaf litter and insect cadavers, increasing the amount of inorganic nitrogen released into the soil [51]. In soil ecosystems, this organic nitrogen is converted to ammonia by a number of ammonifying bacteria such as *Bacillus*, *Pseudomonas*, and *Streptomyces*, after which bacteria, such as *Nitrobacter* and *Nitrosomonas*, are then able to oxidize ammonia into nitrate [52]. Soil fungi also play a key role in the decomposition of insect cadavers and release of organic nitrogen. In the case of insect cadavers and insect frass, extracellular fungal hydrolytic enzymes, such as proteases and chitinases, are capable of breaking down organic nitrogen and converting it into a form useable by plants [53].

### 5.2. Potential Role of Myccorhizal Fungi in Insect Nitrogen Transfer

Root-associated fungi have long been studied for the benefits they provide through their symbiotic relationship with plants. A particular focus is placed on the ubiquitous mycorrhizal fungi that form an internal association with plant roots. These symbiotic fungi are able to increase the overall fitness of their plant partners through an increase in the uptake of critical nutrients, such as nitrogen [54]. These fungi provide nutrients by effectively increasing the surface area of the plant roots and by mobilizing nitrogen [55]. In return, the plant provides the fungus with plant-synthesized carbohydrates, typically in the form of monosaccharides [56].

Mycorrhizal fungi are able to scavenge important soil nutrients, some of which are transferred to plants during internal root colonization [57]. The nutrients that are scavenged can be from any decaying organic matter; however, some mycorrhizal fungi transfer nitrogen from insect cadavers to their plant host. For example, the ectomycorrhizal basidiomycete, *Laccaria bicolor*, was found to harvest nitrogen bound in insect cadavers and subsequently furnish plant roots with this scavenged nitrogen [58]. Several mycorrhizal species are decomposers of organic detritus in the soil [59]. These fungi are able to actively decompose organic matter and transfer useable inorganic nitrogen to plant roots. In this case, the mycorrhizae are able directly to break down insect cadavers or frass in order to obtain nitrogen that can be exchanged within the plant. 

The exchange of nitrogen between mycorrhizal fungi and plant roots is mediated internally by the formation of a nutrient transfer interface. Embedded within this interface are transport proteins that facilitate the movement of phosphorous, nitrogen, and carbon between the two symbionts [60]. This has been observed most prominently in arbuscule mycorrhizal fungi when promoter GUS (β-glucuronidase) fusion has been used to identify the location and expression of fungal and plant transporters within arbuscule-containing cells [61]. These include ammonium and nitrate transporters, such as *gmAMT4* and *MtGim*, respectively [62,63], as well as the fungal phosphate transporters *GmosPT* and *GvPT* and the sugar transporter *Mtst1* [64,65].

The reciprocal nature of this symbiotic partnership has been elucidated by tracking the translocation of nitrogen, phosphorous, and sugars at the plant/fungal interface [56,66]. When the plant is provided with high levels of carbon, there is a greater transfer of nutrients from the fungus to the plant, indicating that the movement of carbon, from the plant to the fungus, is an essential component of nutrient transfer in plant/fungal symbioses [67]. This partnership increases plant productivity and overall plant health. Plants that are able to form complex and symbiotic relationships with soil fungi are stronger competitors with respect to gaining access to soil nutrients such as nitrogen. 

## 6. Endophytic Insect Pathogenic Fungi

Endophytic fungi are a class of plant symbionts that form intercellular root associations, similar to those formed by the mycorrhizae. Unlike mycorrhizal fungi, however, it was considered that endophytes did not develop a specific nutrient transfer interface once inside the plant; as such, it was initially thought that endophytic fungi were not able to provide nutrients to plant roots [68]. Subsequently, it was found that *Heteroconium chaetospira*, a dark septate endophyte, was able to transfer nitrogen to the roots of cabbage plants [69]. *H. chaetospira* is a saprobe that transfers nutrients to plants which have been acquired from decaying organic matter [70]. Several other examples of nutrient transfer by endophytic fungi are known [71]. Recently a new class of endophytic insect pathogenic fungi (EIPF) has been elucidated, which is able to transfer nitrogen obtained from the insects that they infect directly to their plant symbionts [4].

*Metarhizium* and *Beauveria* are examples of insect pathogenic fungi that have recently been discovered to have endophytic capabilities [72,73]. Historically, research on these fungi focused on their insect pathogenicity and their use as biological control agents. These fungi are broad range insect pathogens, and are able to infect over 200 insect species [74]. Why would insect pathogenic fungi possess the ability to colonize plant roots? Work done on *Metarhizium* *robertsii* has shown that this EIPF is able to acquire nitrogen from the insects it infects and, through endophytic association, transfers the insect-derived nitrogen to plants [4]. This finding represents a remarkable adaptation by which plants are able to utilize insects in the soil as a nitrogen source, albeit through a fungal partner.

**Figure 1:**
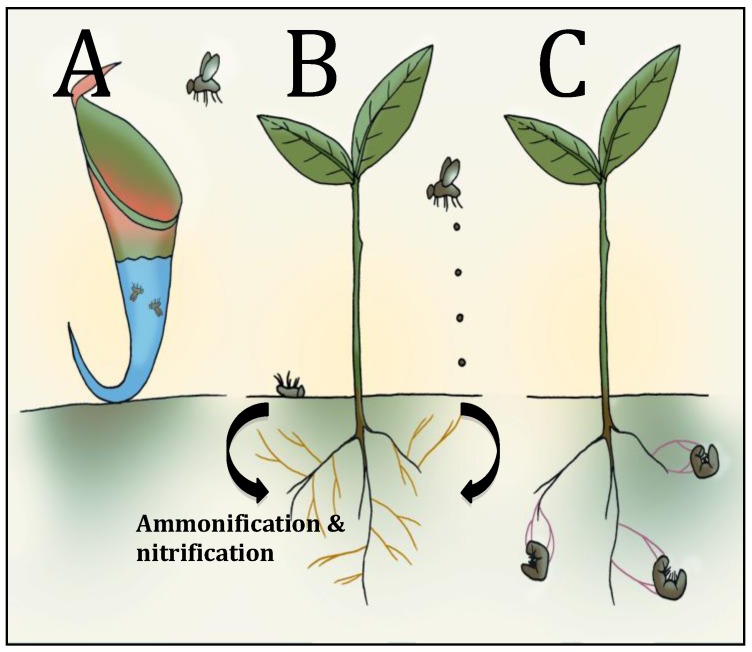
Representation of three major routes by which plants are able to obtain insect bound nitrogen. (**A**) Carnivorous plants capture and digest insects and acquire insect derived nitrogen. Shown is a depiction of a pitcher plant where insects are captured in a pitfall trap containing fluid and are subsequently digested. (**B**) Organic nitrogen within insects can be made available to plants from insect cadavers and from insect frass. Insect-derived nitrogen is microbially converted by ammonification and nitrification and made directly available to plants or, translocated to plants through a symbiotic association with mycorrhizal fungi (shown in yellow). (**C**) Plants are able to acquire insect derived nitrogen through a symbiotic association with EIPF. EIPF (shown as purple) infect and kill insects in the soil and insect-derived nitrogen is translocated directly to plants.

Root feeding insects represent a substantial loss of nitrogen to the plant through leaf and root material as well as through root exudates [10]. This nitrogen loss, if not balanced, could have detrimental effects on plant growth and development [13]. The partnership between an EIPF and a plant could allow plant reacquisition of nitrogen previously lost through below ground insect herbivory (Figure 1).

## 7. Evolutionary Implication of Nitrogen Transfer by Fungal Symbionts

The majority of land plants are able to form intimate associations with soil fungi, either mycorrhizae or endophytes. What is intriguing about these associations is that these fungi are from disparate phylogenetic lineages [75,76]. This suggests that these fungi have undergone convergent evolution with respect to plant symbiosis. The intimacy of this association was potentially driven by fungal acquisition of plant-fixed carbon. Plants can fix carbon dioxide and are a carbon reservoir, a valuable commodity to fungi [77]. Plants would allow carbon exchange to a partner that could provide nutrients. Nitrogen is a valuable commodity to plants and symbionts that provide nitrogen may have access to plant carbon. In this review we considered insects as a nitrogen reservoir and plant adaptations that allow access to insect nitrogen. Several examples of fungal symbionts that provide nitrogen to plants were outlined, and the implication is that the plant host may be driving the evolution of endophytism. 

The traditional paradigm concerning the population structure of insect pathogenic fungi has focused on the insect host as the driving force. However, the fact that some pathogenic fungi are endophytes (*Beauveria* and *Metarhizium*) suggests that plant host symbiosis may be a major evolutionary influence [77]. Insect pathogenic fungi that are incapable of forming plant associations would be more likely to evolve insect host specificity and, in this case, fungal evolution would be driven most influentially by the range of insect hosts. This type of specificity would provide an advantage for these insect pathogenic fungi under certain conditions. However, EIPF have a broad insect host range, which would be advantageous to the plant as well as to the fungus, *i.e.*, a larger insect nitrogen reservoir to use in trading for carbon. This implies that the plant host is driving the evolution of the broad range EIPF, since it would be beneficial for the plant to associate with fungal partners that are able to exploit a broad range of insects.

## 8. Conclusions

Nitrogen cycling in the soil has largely ignored insects as a nitrogen reservoir. Many species of plants have evolved adaptations by which to exploit nitrogen present in insects. Carnivorous plants survive, almost exclusively, from nitrogen found in insects [78]. While carnivorous plants are one of the best known examples of a plant adaptation in order to access insect nitrogen, plants that are incapable of enzymatic/mechanical digestion have developed novel fungal partnerships in order to exploit insects as a nitrogen source. By partnering with mycorrhizal fungi, plants can gain access to nitrogen, of which one form is derived from decomposing insects and insect frass. Another, more active, symbiosis for the acquisition of insect nitrogen is through EIPF, such as *Metarhizium* or *Beauveria*. EIPF are able to infect live insects and translocate insect-derived nitrogen to their plant partners, potentially in exchange for plant-synthesized carbon, as has been found for mycorrhizal fungi. These associations have theoretically evolved to satisfy fungal acquisition of carbon, and have driven EIPF to a broad insect host range that provides plants with the largest potential source of nitrogen. These symbioses have potentially driven the evolution of soil fungi (e.g., mycorrhizal and EIPF) in a manner that allows non-carnivorous plants to reacquire the nitrogen lost through insect herbivory.
